# Fibrous Mesoporous Silica KCC-1 Functionalized with 3,5-Di-tert-butylsalicylaldehyde as an Efficient Dispersive Solid-Phase Extraction Sorbent for Pb(II) and Co(II) from Water

**DOI:** 10.3390/nano16010058

**Published:** 2025-12-31

**Authors:** Sultan K. Alharbi, Yassin T. H. Mehdar, Manal A. Almalki, Khaled A. Thumayri, Khaled M. AlMohaimadi, Bandar R. Alsehli, Awadh O. AlSuhaimi, Belal H. M. Hussein

**Affiliations:** 1 Chemistry Department, Faculty of Science, Taibah University, Madinah Al-Munawaroh 41321, Saudi Arabia; sbdrani@taibahu.edu.sa (S.K.A.); ymehdar@taibahu.edu.sa (Y.T.H.M.); mmalky@taibahu.edu.sa (M.A.A.); kthumairi@taibahu.edu.sa (K.A.T.); bshle@taibahu.edu.sa (B.R.A.); belalhussein102@yahoo.com (B.H.M.H.); 2 Ministry of Education, Director General of Education, Medinah Al-Munwwarah 42314, Saudi Arabia; khaled-mohaimadi@hotmail.com; 3 Chemistry Department, Faculty of Science, Suez Canal University, Ismailia 41522, Egypt

**Keywords:** 3,5-di-tert-butylsalicylaldehyde, adsorption kinetics, isotherms, solid-phase microextraction, heavy metal removal

## Abstract

The accurate determination of trace metals in aqueous matrices necessitates robust sample preparation techniques that enable selective preconcentration of analytes while ensuring compatibility with subsequent instrumental analysis. Dispersive solid-phase extraction (d-SPE), a suspension-based variant of conventional solid-phase extraction (SPE), facilitates rapid sorbent–analyte interactions and enhances mass transfer efficiency through direct dispersion of the sorbent in the sample solution. This approach offers significant advantages over traditional column-based SPE, including faster extraction kinetics and greater operational simplicity. When supported by appropriately engineered sorbents, d-SPE exhibits considerable potential for the selective enrichment of trace metal analytes from complex aqueous matrices. In this work, a fibrous silica-based chelating material, DSA-KCC-1, was synthesized by grafting 3,5-Di-tert-butylsalicylaldehyde (DSA) onto aminopropyl-modified KCC-1. The dendritic KCC-1 scaffold enables fast dispersion and short diffusion pathways, while the immobilized phenolate–imine ligand introduces defined binding sites for transition-metal uptake. Characterization by FTIR, TGA, BET, FESEM/TEM, XRD, and elemental analysis confirmed the successfulness of functionalization and preservation of the fibrous mesostructured. Adsorption studies demonstrated chemisorption-driven interactions for Pb(II) and Co(II) from water, with Langmuir-type monolayer uptake and pseudo-second-order kinetic behavior. The nano-adsorbent exhibited a markedly higher affinity for Pb(II) than for Co(II), with maximum adsorption capacities of 99.73 and 66.26 mg g^−1^, respectively. Integration of the DSA-KCC-1 nanosorbent into a d-SPE–ICP-OES workflow enabled the reliable determination of trace levels of the target ions, delivering low limits of detection, wide linear calibration ranges, and stable performance over repeated extraction cycles. Analysis of NIST CRM 1643d yielded results in good agreement with the certified values, while the method demonstrated high tolerance toward common coexisting ions. The combined structural features of the KCC-1 support and the Schiff-base ligand indicate the suitability of DSA-KCC-1 for d-SPE workflows and demonstrate the potential of this SPE format for selective preconcentration of trace metal ions in aqueous matrices.

## 1. Introduction

The persistence of heavy-metal contaminants in aquatic environments poses a continuing environmental and public-health challenge due to their non-biodegradable nature, high toxicity, and strong tendency for bioaccumulation and biomagnification [1,2]. Continuous anthropogenic inputs from mining, electroplating, pigment and battery manufacturing, and industrial effluent discharge have resulted in the widespread occurrence of metal ions in surface water, groundwater, and industrial wastewater, frequently at trace or ultra-trace concentrations [1]. Among these pollutants, Pb(II) and Co(II) are of particular concern owing to their extensive industrial use, environmental persistence, and repeated detection in aquatic systems [2].

Stringent drinking-water guideline values have been established by the World Health Organization (WHO), with a maximum residual limit of 0.01 mg L^−1^ for Pb(II) and 0.07 mg L^−1^ for Co(II) [3]. The frequent presence of these ions at concentrations approaching or below regulatory limits highlights the need for analytical strategies that combine high sensitivity and selectivity with strong resistance to matrix interference.

Despite the availability of advanced instrumental techniques capable of detecting metals at sub-ng L^−1^ concentrations, sample pre-treatment remains a critical step in the analysis of trace metals, particularly for analyte preconcentration and matrix separation. Conventional methods like liquid–liquid extraction, cloud-point extraction, chemical precipitation, and classical solid-phase extraction, are often limited by high solvent consumption, slow mass transfer, and insufficient selectivity [4,5,6,7,8,9,10]. These constraints have stimulated growing interest in dispersed solid-phase extraction (dSPE), which offers rapid analyte–sorbent interaction, operational simplicity, high enrichment factors, and reduced solvent usage [11,12,13]. The effectiveness of dSPE is therefore governed by the physicochemical properties of the sorbent, which must provide accessible surface area, efficient diffusion pathways, and strong chelating functionalities for selective capture of metals.

Among next-generation silica sorbents, KCC-1 (KAUST Catalysis Center-1) has gained considerable attention. First introduced by Polshettiwar and Basset at the KAUST Catalysis Center [14], KCC-1 belongs to the class of fibrous or dendritic mesoporous silica nanoparticles (FMSNs/DMSNs). Unlike ordered mesoporous silicas such as MCM-41 and SBA-15, KCC-1 exhibits a three-dimensional, radially oriented fibrous morphology, which provide exceptionally high external surface area, large pore volume, hierarchical accessibility, and efficient solvent penetration [14,15,16,17,18]. These structural characteristics promote enhanced mass-transfer kinetics and underpin the versatility of KCC-1 in catalysis, adsorption, sensing, and environmental remediation. Nevertheless, pristine KCC-1 lacks inherent metal-ion specificity; thus, surface functionalization with tailored coordination sites is employed to enhance selectivity.

The surface chemistry of KCC-1 closely resembles that of conventional silica and other nanostructured siliceous materials, rendering it a versatile platform for coordination-site engineering. KCC-1 possesses a high density of surface silanol (Si–OH) groups, which serve as reactive anchoring sites for post-synthetic silanization. This well-established modification strategy enables the stable covalent immobilizations of organic ligands bearing well-defined chelating functionalities, thereby allowing tailored metal-binding behavior. Through such modification, the local coordination environment at the solid–solution interface can be systematically tuned in terms of donor identity (e.g., N, O, S), denticity, and electronic properties, thereby governing metal–ligand interactions in accordance with established coordination-chemistry principles. As a result, functionalization strategies routinely applied to ordered mesoporous silicas can be directly translated to KCC-1, allowing the achievement of controlled metal-ion recognition without compromising its fibrous architecture [19,20,21,22,23].

Consistent with this framework, numerous studies have demonstrated that donor-rich functionalization of KCC-1 effectively modulates its metal-binding behavior. Marjani and Mohammadi [24] reported tetrasulfide-functionalized KCC-1 (TSF-KCC-1), in which soft sulfur donor atoms preferentially coordinate Hg(II) through soft–soft interactions. Soltani et al. [25] incorporated *N*-methylimidazolium functionalities into KCC-1, producing MI-Cl-KCC-1 with high affinity for Cr(VI) oxyanions. Zarei et al. [26] prepared a triamino-functionalized KCC-1/chitosan–oleic acid composite that exploits multidentate nitrogen donors for Pb(II) coordination, while Soltani et al. [27] fabricated a carboxyl-functionalized KCC-1 (COOH-KCC-1) hybrid exhibiting strong binding toward Cd(II) through oxygen–donor interactions. Extending these concepts to analytical chemistry, Behbahani and co-workers [28] employed amine-functionalized KCC-1 in a dispersive micro-solid-phase extraction (d-µ-SPE) workflow for trace-level determination of Pb(II) and Cd(II).

From a coordination-chemistry perspective, salicylaldehyde-based Schiff bases represent particularly attractive modifiers owing to their straightforward synthesis, ease of immobilization, and well-defined N, O-donor binding motifs. Metal coordination proceeds via cooperative interaction of the imine nitrogen and phenolate oxygen atoms, resulting in stable chelate complexes. When immobilized on mesoporous silica supports, such ligands have been shown to enhance adsorption capacity, improve uptake kinetics, and allow repeated reuse while maintaining structural integrity [29,30]. SBA-15-type mesoporous silica modified with a bis-salicylaldehyde Schiff base (SBA-DSA) was developed for the removal of Pb(II) from water was found to exhibit a maximum adsorption capacity of 60.9 mg g^−1^ [31]. In related work, two thiazole–Schiff base–functionalized mesoporous silica adsorbents were synthesized using SBA-15 as the support material. The silica surface was first modified with either aminopropyl or [2-aminoethylamino]propyl groups, followed by Schiff base condensation with 2-thiazolecarboxaldehyde. This approach produced two structurally distinct adsorbents, exhibiting theoretical maximum Pb(II) adsorption capacities of 149.1 and 99.7 mg g^−1^, respectively [29].

This investigation employs the established covalent immobilization protocols to functionalize the dendritic fibrous silica framework of KCC-1 with the sterically hindered Schiff base ligand 3,5-di-tert-butylsalicylaldehyde (DSA), to fabricate a novel chelate-modified sorbent (DSA–KCC-1) specifically engineered for dSPE applications. The incorporated DSA moiety imparts a robust, sterically protected phenolate–imine coordination environment conducive to strong and selective binding of divalent metal cations, whereas the characteristic fibrous morphology of KCC-1 facilitates rapid dispersibility, minimized diffusional resistance, and maximal exposure of chelating sites. This integrated design confers marked improvements in selectivity, adsorption kinetics, and overall extraction performance over conventional adsorbents, including metal–organic frameworks, carbon nanotubes, graphene-based materials, layered clays, polymeric resins, and amorphous or non-fibrous silicas [32,33,34,35,36,37,38,39]. Beyond demonstrating fundamental nanochelation principles, the DSA–KCC-1 material serves as a practical analytical platform, enabling sensitive and reliable dSPE-based preconcentration and determination of low concentrations of Pb(II) and Co(II) in complex aqueous matrices, thereby facilitating the broader integration of advanced dSPE methodologies into routine environmental monitoring and quality assurance protocols.

## 2. Materials and Methods

### 2.1. Chemical Reagents

Deionized water (resistivity > 18.2 MΩ·cm) was obtained from a Milli-Q system (Elix Millipore, Molsheim, France). All chemicals were used as received without further purification. 3-Aminopropyltriethoxysilane (APTES), tetraethyl orthosilicate (TEOS), hexadecyltrimethylammonium chloride (CTAC), urea, cyclohexane, amyl alcohol, ethanol and 3,5-di-tert-butylsalicylaldehyde (DSA) were purchased from Sigma-Aldrich (St. Louis, MO, USA). Sodium hydroxide micropearls (>99%) were obtained from Merck (Darmstadt, Germany). Hydrochloric acid (37%), nitric acid (65%), acetic acid and ammonium hydroxide solution (25%) were sourced from Shanghai Chemical Reagent Co. (Shanghai, China) and employed for the preparation of buffer used in the preparation of standard solutions. Stock solutions of Co(II) and Pb(II) (1000 mg L^−1^ each) were obtained from certified inorganic standard solutions supplied by Inorganic Ventures (Christiansburg, VA, USA).

### 2.2. Instrumentations

Field-emission scanning electron microscopy (FESEM) images were obtained using a Zeiss EM10C instrument operated at 100 kV (Carl Zeiss, Oberkochen, Germany). The average particle size was calculated from these images employing ImageJ software version 1.54 (National Institutes of Health, Bethesda, MD, USA). Transmission electron microscopy (TEM) imaging was performed with a JEM-1400 Flash instrument (JEOL Inc., Peabody, MA, USA). X-ray diffraction (XRD) patterns were recorded on a Rigaku Multiflex diffractometer (Rigaku Corporation, Tokyo, Japan) equipped with monochromated high-intensity Cu Kα radiation (λ = 1.54 Å). Data were collected under ambient conditions in the 2θ range of 0.7–5° at a scan rate of 0.1° min^−1^ (20 kV, 10 mA).

Nitrogen adsorption–desorption isotherms were measured at 77 K using a Micromeritics ASAP 2020 physisorption analyzer (Micromeritics Instrument Corporation, Norcross, GA, USA) to assess pore characteristics. Specific surface areas (S_BET) were derived from the linear portion of the Brunauer–Emmett–Teller (BET) plot, whereas pore size distributions were determined via the Barrett–Joyner–Halenda (BJH) method. Prior to analysis, samples were dispersed on a steel plate and sputter-coated with platinum.

Fourier-transform infrared (FTIR) spectra were acquired with a Thermo Fisher Scientific spectrometer (Waltham, MA, USA). Thermogravimetric analysis (TGA) was conducted using a NEXTA STA200 analyzer (Hitachi High-Tech Corporation, Tokyo, Japan) to monitor mass loss as a function of temperature.

The concentrations of the studied metal ions were quantified by inductively coupled plasma optical emission spectrometry (ICP-OES) employing an Agilent 5900 series instrument (Agilent Technologies Inc., Santa Clara, CA, USA), operated according to the manufacturer’s protocols.

Nonlinear regression analysis (NLRA) of isotherm and kinetic models was performed utilizing the statistical tools in OriginPro 8.6 software (OriginLab Corporation, Northampton, MA, USA).

### 2.3. Synthesis of KCC-1

Pristine KCC-1 were synthesized via a modified sol–gel–hydrothermal method adapted from established literature procedures [24,25,27]. Tetraethyl orthosilicate (TEOS) was employed as the silica precursor in a biphasic cyclohexane–water system, with urea and cetyltrimethylammonium bromide (CTAB) acting as co-structure-directing agents. This approach affords a fibrous silica framework with a high density of surface silanol (Si–OH) groups, providing a chemically accessible platform for subsequent post-synthetic functionalization.

In a typical experiment, urea (0.6 g) and CTAB (0.7 g) were dissolved in deionized water (50 g) in a 200 mL Teflon-lined stainless-steel autoclave and stirred at 298 K for 30 min. A cyclohexane solution of TEOS was then added dropwise over 15 min, leading to the formation of a homogeneous milky emulsion. Subsequently, *n*-amyl alcohol (15 mL) was introduced dropwise under vigorous stirring (500 rpm) for 15 min, followed by continued stirring for an additional 30 min to ensure uniform dispersion. The sealed autoclave was then subjected to hydrothermal treatment at 393 K for 8 h.

After natural cooling to room temperature, the resulting solid was isolated by centrifugation (6000 rpm) and washed successively with deionized water and ethanol (3 × 50 mL each) to remove residual reactants. The material was dried at 363 K for 24 h and calcined in static air at 823 K for 6 h to remove the organic templates, thereby a pristine KCC-1 with an open fibrous morphology and dense surface silanol density, ideally suited for post-synthetic grafting reactions was obtained.

### 2.4. Functionalization of KCC-1 with 3,5-Di-tert-butylsalicylaldehyde (DSA)

The acid-activated KCC-1 (1.00 g) was dispersed in anhydrous toluene (50 mL) in a 250 mL round-bottom flask by ultrasonication for 15 min, followed by the addition of (3-aminopropyl)triethoxysilane (APTES, 1.0 mL). The mixture was refluxed under a dry nitrogen atmosphere for 8 h with continuous stirring to graft aminopropyl groups onto the silica surface. After cooling to room temperature, the solid was collected by centrifugation, washed thoroughly with deionized water and ethanol to remove residual silane species, and dried at 343 K for 24 h to obtain the amine-modified material (AP-KCC-1). The AP-KCC-1 intermediate was subsequently reacted with 3,5-di-tert-butylsalicylaldehyde (DSA) under reflux in anhydrous toluene, affording the Schiff base-functionalized KCC-1 material (DSA-KCC-1) through surface imine formation.

### 2.5. Adsorption Procedure

The adsorption performance of the synthesized DSA-KCC-1 chelator was evaluated by batch adsorption experiments using Co(II) and Pb(II) ions as representative examples to examine the effects of solution pH, adsorption kinetics, and equilibrium isotherms. In a typical experiment, 10 mg of adsorbent (100 mg L^−1^) was added to 100 mL of metal ion solution prepared in 1.0 M ammonium acetate buffer. The solution pH was adjusted within the investigated range, and the initial metal concentration was varied as required. The suspension was agitated at 200 rpm to ensure uniform dispersion and minimize mass-transfer limitations. At selected time intervals, 5 mL aliquots were withdrawn, centrifuged, and the supernatant was acidified with 2% (*v*/*v*) HNO_3_ prior to ICP-OES analysis. The removal efficiency (*η*, %) and equilibrium adsorption capacity (*q_e_*, mg g^−1^) were calculated using Equations (1) and (2), respectively.

(1) $$\eta (\%)=\left(\frac{C_{i}-C_{e}}{C_{i}}\right)\times 100$$

(2) $$q_{e}=\frac{(C_{i}-C_{e})\times V}{m}$$

where *C_i_* and *C_e_* are the initial and equilibrium concentrations of the metal ion (mg/L), *V* is the volume of the solution (L), and *m* is the mass of the adsorbent (g).

### 2.6. dSPE Procedure

The optimized dSPE procedure was uniformly applied to calibration standards, procedural blanks, and real water samples. Aliquots of 50.0 mL—comprising either calibration standards, ultrapure-water blanks, or samples—were transferred into 100 mL centrifuge tubes. Subsequently, 50 mg of the DSA-KCC-1 sorbent was added, and the suspension was vortex-mixed at 3000 rpm for 20 min to ensure homogeneous dispersion and effective adsorption of Pb(II) and Co(II) ions. Subsequently, the sorbent was separated by centrifugation at 4000 rpm for 5 min, and the supernatant was discarded. Desorption of the chelated metal ions was achieved by redispersing the DSA-KCC-1 phase in 5.0 mL of 1.0 M HNO_3_ with vortex agitation for 5 min. The resulting eluate was analyzed by ICP-OES after appropriate dilution to reduce the nitric acid concentration.

All extractions were performed in triplicate, and calibration standards and procedural blanks were processed concurrently with each analytical batch to ensure method consistency and accurate quantification. The desorption efficiency (*η*_des_) was calculated using Equation (3).

(3) $$\eta_{\mathrm{des}}=\left(\frac{C_{\mathrm{des}}}{C_{\mathrm{ads}}}\right)\times 100$$

where $C_{\mathrm{des}}$ and $C_{\mathrm{ads}}$ are the desorbed and adsorbed concentrations of the metal ions, respectively.

## 3. Results

### 3.1. Synthesis and Characterization of KCC-1 and DSA-KCC-1

The hydrothermal-assisted sol–gel approach was found to reproducibly generate KCC-1 with a characteristic fibrous dendritic architecture. This type of morphology is widely associated with high surface areas and a substantial population of surface silanol groups, features that are generally regarded as favorable for post-synthetic modification of silica-based materials. In the present work, amine functionalization was therefore pursued using a post-grafting strategy based on APTES, in which hydrolyzed silane species are expected to interact with surface silanol groups on KCC-1. The formation of AP-KCC-1 is accordingly interpreted to arise from the establishment of Si–O–Si linkages, as inferred from the combined characterization results.

**Figure 1:**
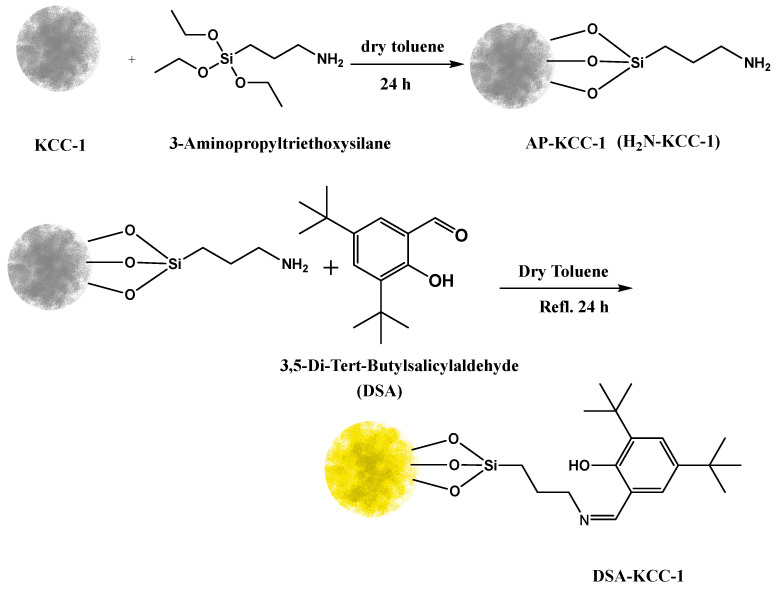
General reaction processes preparation of DSA-KCC-1, showing the associated color changes from white (pristine KCC-1) to yellow Schiff base (DSA-KCC-1).

Subsequent Schiff-base modification was carried out through condensation between the primary amine groups of AP-KCC-1 and the aldehyde functionality of the DSA ligand, yielding an immobilized chelating framework. The formation of imine (C=N) bonds was supported by spectroscopic evidence and was accompanied by the appearance of a yellow heterogeneous product. As illustrated in Figure 1, the proposed modification pathway involves sequential silanization followed by imine formation. The progression of these post-synthetic modification steps was also qualitatively reflected by distinct color changes observed after each key synthetic stage.

The presence of tert-butyl substituents within the DSA framework may provide additional advantages, as such bulky groups can introduce steric shielding and locally hydrophobic environments around the donor atoms. These features have been reported to enhance the stability and coordination behavior of immobilized Schiff-base ligands [40,41], thereby supporting the design rationale of DSA-KCC-1 as a potentially effective nanosorbent.

**Figure 2:**
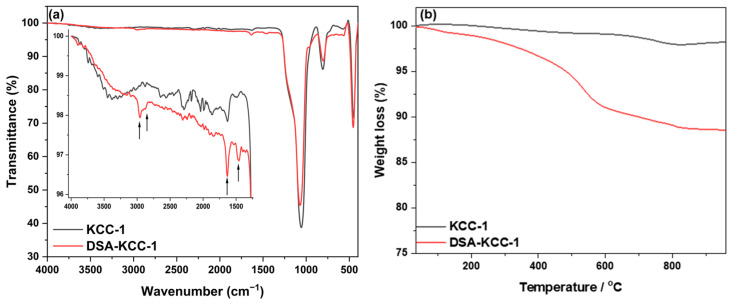
The FTIR spectra (**a**) and thermogram (**b**) of calcined KCC-1 and DSA-KCC-1.

The successful functionalization of KCC-1 was confirmed by FTIR spectroscopy (Figure 2a). Pristine KCC-1 displayed broad O–H stretching (3200–3600 cm^−1^), water-bending (~1620 cm^−1^), and characteristic Si–O–Si vibrations at 1235, 1100, 975, 800, and 455 cm^−1^, consistent with reported mesoporous silica materials [10,42,43]. In contrast, DSA-KCC-1 showed additional C–H stretching and bending bands at 2926, 2854, and 1490 cm^−1^, as well as a Si–C vibration at ~760 cm^−1^, confirming APTES grafting and DSA immobilization.

Thermogravimetric analysis (TGA, Figure 2b) provides clear evidence of the differences in thermal behavior and organic loading between pristine and functionalized materials. Calcined KCC-1 exhibits negligible mass loss above 200 °C, confirming its high thermal stability and the effective removal of residual organic species during calcination. In contrast, DSA-KCC-1 shows three distinct weight-loss events centered at approximately 180, 238, and 433 °C, with a total mass loss of 12.00%, indicating substantial incorporation of the organic ligand [44].

The initial weight loss below 200 °C is attributed to the desorption of physisorbed water and residual solvent molecules. The second decomposition step, occurring around 238 °C, corresponds to the thermal degradation of the Schiff-base functionality, primarily involving cleavage of the imine (–C=N–) linkage. The third event at higher temperature (~433 °C) is associated with the decomposition of covalently bound organosilane chains, accompanied by condensation of surface silanol (Si–OH) groups. Notably, the persistence of organic mass loss at elevated temperatures supports the robust covalent anchoring of the Schiff-base ligand onto the silica framework rather than weak physisorption.

Elemental analysis (EA) further substantiates the successful grafting of the DSA ligand onto the KCC-1 surface via Schiff-base condensation with aminopropyl groups. As summarized in Table 1, the experimentally determined carbon, hydrogen, and nitrogen contents are in close agreement with the calculated values for the proposed surface-bound structure. The derived atomic ratios are consistent with the theoretical composition of a C_18_H_28_N_1_O_1_ moiety, corresponding to an organic loading of approximately 12 wt%, in excellent agreement with the TGA results. The slightly elevated hydrogen content can reasonably be attributed to residual surface silanol groups and physisorbed moisture, which are commonly observed in silica-based materials.

Collectively, the TGA and EA results provide complementary and consistent evidence for the successful formation of a stoichiometric 1:1 Schiff-base linkage between the DSA ligand and the aminofunctionalized KCC-1 surface.

**Figure 3:**
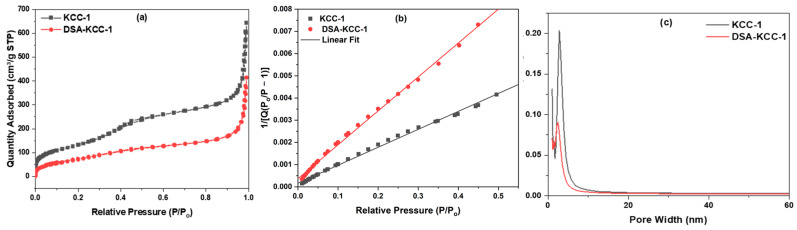
(**a**,**b**) N2 adsorption/desorption isotherms of pristine KCC-1 and DSA-KCC-1 and (**c**) the BJH pore size distribution curves of the samples.

Nitrogen adsorption–desorption isotherms (Figure 3) of pristine KCC-1 and DSA-KCC-1 displayed type IV curves with H1-type hysteresis loops, as classified by IUPAC, indicative of well-defined mesoporous structures with cylindrical pores. The pristine KCC-1 exhibited a high Brunauer–Emmett–Teller (BET) surface area of 530 m^2^ g^−1^, which decreased to 380 m^2^ g^−1^ after functionalization. Correspondingly, the Barrett–Joyner–Halenda (BJH) pore volume and average pore diameter reduced from 1.02 to 0.67 cm^3^ g^−1^ and from 2.85 to 2.53 nm, respectively. These reductions are attributable to partial occupation of the mesoporous channels by the grafted organic moieties, a common observation in surface-modified silica materials [44]. The detailed textural properties are summarized in Table 2.

The distinctive fibrous morphology of KCC-1 arises from hydrothermal treatment, which facilitates extensive silica condensation and yields enhanced structural ordering. Subsequent template removal by calcination produces a dendritic architecture comprising open, radially arranged, and highly accessible mesoporous channels [33]. This unique structure differentiates KCC-1 from conventional ordered mesoporous silicas (e.g., MCM-41 or SBA-15) and imparts superior mass transfer kinetics, rendering it particularly effective for adsorption, solid-phase extraction, and related applications [45].

**Figure 4:**
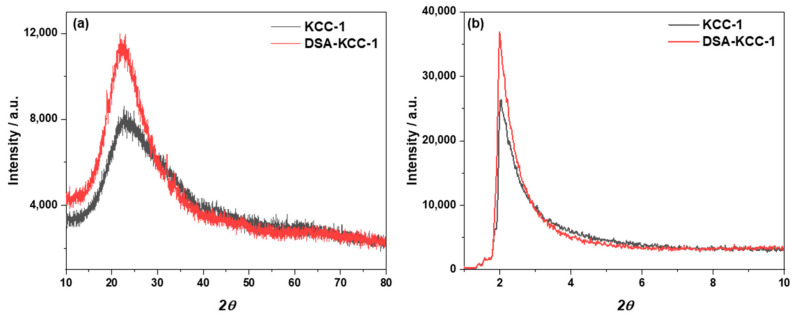
Long angle XRD (**a**) and small angle XRD (**b**) for KCC-1 and DSA-KCC-1.

The long-angle XRD patterns (Figure 4a) of KCC-1 and DSA-KCC-1 show a broad diffraction band centered at approximately 23° (2θ), consistent with the amorphous nature of the silica framework. No additional crystalline reflections are observed after surface functionalization, indicating that the fibrous structure of KCC-1 remains unchanged. In the low-angle region (Figure 4b), pristine KCC-1 exhibits a distinct diffraction peak at 2θ ≈ 2.04°, reflecting the presence of an ordered mesoporous architecture. This reflection is preserved in DSA-KCC-1 with a slight shift to lower angle (2θ ≈ 1.99°). The observed shift reflects minor changes in the mesoporous lattice associated with DSA incorporation, while the overall mesostructural order of KCC-1 is maintained.

**Figure 5:**
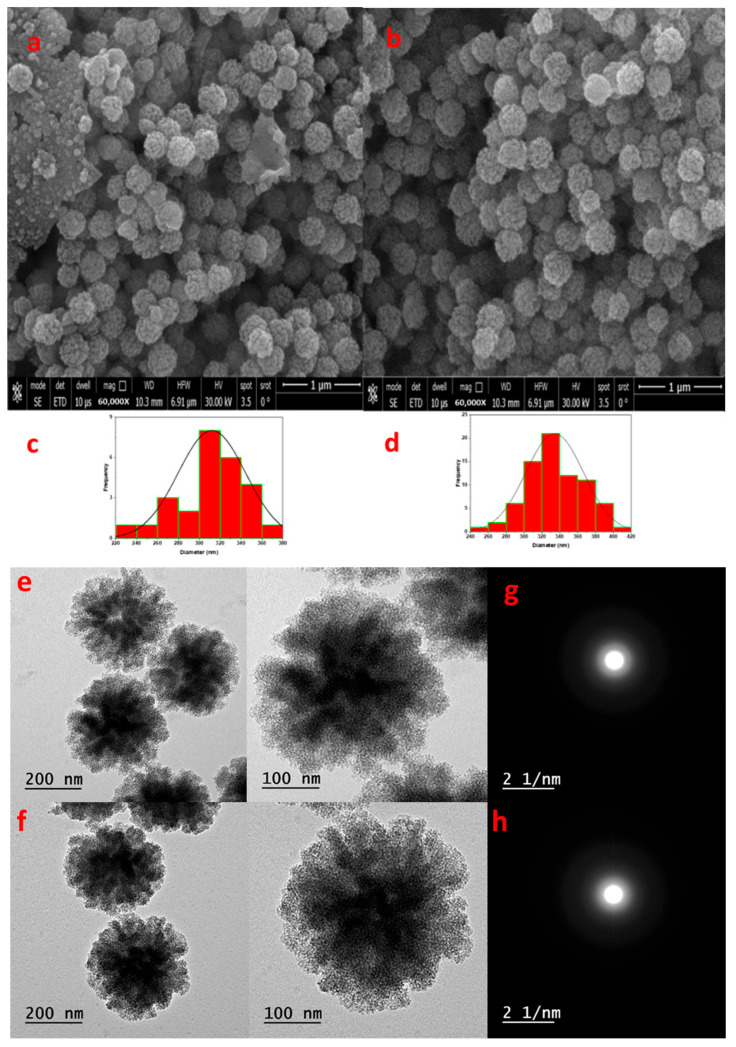
(**a**,**b**) FESEM images, (**c**,**d**) Histogram, (**e**,**f**), and (**g**,**h**) SAED of KCC-1 and DSA-KCC-1.

FESEM and TEM images (Figure 5) show that both pristine KCC-1 and DSA-KCC-1 maintain a uniform spherical morphology (300–400 nm) with a well-defined fibrous dendritic architecture. The DSA-KCC-1 sample exhibits slightly denser fiber packing, consistent with surface functionalization by organic moieties, while preserving the original structural integrity of the KCC-1 framework.

**Figure 6:**
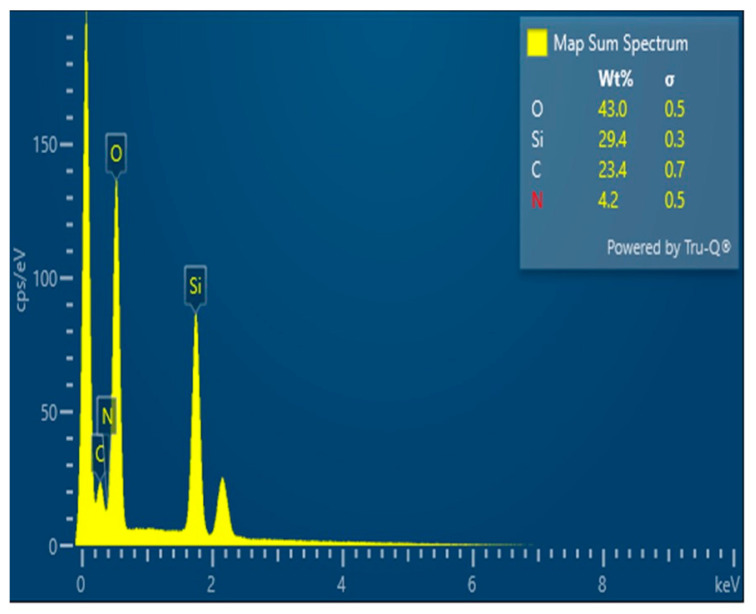
EDX of DSA-KCC-1.

EDX analysis (Figure 6) confirms the presence of Si and O as the main framework elements and reveals distinct C and N signals in DSA-KCC-1. The appearance of carbon and nitrogen, absent in pristine KCC-1, provides direct evidence for successful grafting of the organic molecules onto the silica surface.

### 3.2. Adsorption Performance

A systematic evaluation of adsorption performance was conducted to assess the suitability of DSA-KCC-1 for DSPE. The pH dependence, adsorption kinetics, and equilibrium isotherms were investigated to provide mechanistic insight into ligand-directed metal binding, surface affinity, and mass-transfer contributions, all of which directly govern extraction efficiency in DSPE workflows.

#### 3.2.1. Effect of pH

**Figure 7:**
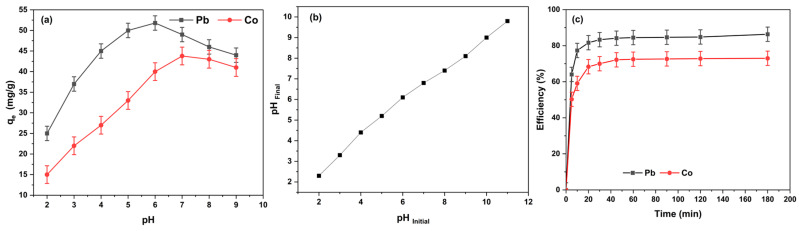
Effect of pH (**a**) and contact time (**c**) on the adsorption of Pb(II) and Co(II) on the DSA-KCC-1 sorbent. (Initial concentration = 60 mg L^−1^, contact time = 60 min, the adsorbent dosage = 0.1 g, temperature = 25.5 °C). (**b**): The Point of Zero Charge (pH_PZC_) of DSA-KCC-1.

The solution pH plays a critical role in the adsorption of Pb(II) and Co(II) onto DSA–KCC-1, as it controls both the speciation of the metal ions in solution and the surface charge/protonation state of the grafted Schiff-base ligand. Figure 7a reveals a marked increase in adsorption capacity for both metal ions as the pH rises from 2.0 to approximately 6.0–7.0. This trend is primarily driven by the progressive deprotonation of the phenolic hydroxyl and imine groups in the surface-bound DSA ligand, which enhances the availability of oxygen and nitrogen donor atoms for coordination with metals. At higher pH values, the formation of stable inner-sphere complexes is favored.

The point of zero charge (pH_PZC_) of DSA–KCC-1 was found to be ca. 5.5 (Figure 7b). At pH < pH_PZC_, the adsorbent surface is positively charged due to protonation of the functional groups, leading to electrostatic repulsion of the cationic metal species and consequently low uptake. In contrast, at pH > pH_PZ_C, the surface becomes negatively charged, promoting electrostatic attraction that synergistically supports specific coordination binding.

In highly acidic media (pH < 4.0), protonation of the donor sites significantly impairs their coordinating ability, while cationic repulsion further suppresses adsorption. The highest removal efficiencies were attained at pH 6.0 for Pb(II) and pH 7.0 for Co(II), where the ligand is sufficiently deprotonated and the metals exist predominantly as free hydrated cations. A modest decline in adsorption beyond pH 7–8 can be ascribed to the incipient precipitation of metal hydroxides, reducing the concentration of soluble ionic species available for binding.

#### 3.2.2. Adsorption Kinetics

Kinetic modeling was performed to elucidate the rate-determining steps and adsorption mechanisms of Pb(II) and Co(II) uptake onto DSA-KCC-1, which is critical for dispersive solid-phase extraction (dSPE) applications requiring rapid sorbent–analyte interactions. Time-dependent adsorption data were fitted using non-linear regression to the pseudo-first-order (PFO) model [46], pseudo-second-order (PSO) model [47], Elovich model [48], and intraparticle diffusion (IPD) model [49]. The corresponding kinetic equations and model parameters are summarized in Table 3.

As shown in Figure 7c, the adsorption was rapid in the initial stage, reaching near-equilibrium within ~60 min for both ions, attributable to efficient mass transfer through the open dendritic fibrous channels of KCC-1 and high accessibility of the immobilized Schiff-base chelating sites.

Nonlinear fitting demonstrated superior performance of the PSO model, evidenced by closest agreement between calculated and experimental *q_e_* values and the lowest RMSE (Table 3). This indicates chemisorption as the rate-limiting step, consistent with coordination of Pb(II) and Co(II) to the phenolate–imine donor moieties on DSA-KCC-1.

**Figure 8:**
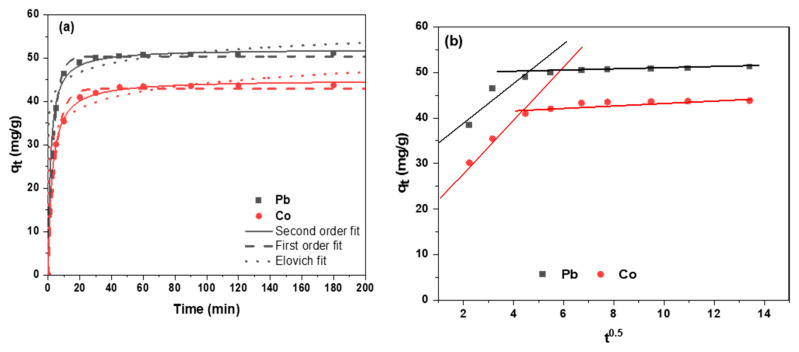
Adsorption kinetic experimental (**a**) and IPD model (**b**) of metal ions on DSA-KCC-1. (Initial concentration = 60 mg/L; contact time = 60 min, the adsorbent dosage = 0.1 g, pH = 6–7, temperature = 25.5 °C).

Intraparticle diffusion (IPD) plots (Figure 8b) displayed two distinct linear segments, evidencing multi-step kinetics: an initial rapid film diffusion phase (external mass transfer) followed by slower intraparticle diffusion within the fibrous silica channels. The non-zero intercept (C > 0) confirms that intraparticle diffusion is not the sole rate-controlling mechanism, with boundary layer effects also contributing significantly.

#### 3.2.3. Adsorption Isotherms

**Figure 9:**
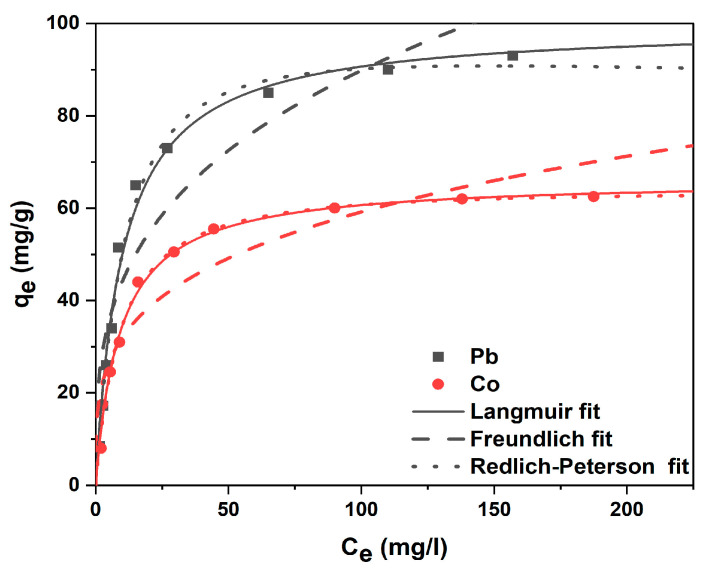
Adsorption isotherm experimental of metal ions on DSA-KCC-1 and nonlinear fitting models.

The equilibrium adsorption of Co(II) and Pb(II) on DSA-KCC-1 was analyzed using the Langmuir [50], Freundlich [51], and Redlich–Peterson (R–P) [52] models. The fitted parameters and nonlinear isotherms are summarized in Table 4 and Figure 9, respectively.

For both metal ions, the Langmuir model provides the best fit to the experimental data, as indicated by the lowest RMSE values and R–P β parameters close to unity. This behavior is characteristic of site-specific monolayer adsorption governed by inner-sphere metal–ligand coordination at energetically equivalent chelating sites. The corresponding Langmuir maximum adsorption capacities reached 99.73 mg g^−1^ for Pb(II) and 66.26 mg g^−1^ for Co(II), reflecting strong affinity toward the immobilized DSA ligand.

In contrast, the higher RMSE values obtained for the Freundlich model suggest that heterogeneous multilayer adsorption is not dominant. Although the fibrous silica framework introduces some structural heterogeneity, metal uptake is mainly controlled by ligand-directed complexation rather than nonspecific surface interactions. The intermediate fit of the R–P model, with β values approaching unity, further supports the predominance of Langmuir-type adsorption.

### 3.3. Analytical Method Performance (Figures of Merit)

The synthesized nanochelator was evaluated as a dSPE sorbent for the extraction and preconcentration of Pb(II) and Co(II) prior to ICP–OES determination. Method performance was assessed using matrix-matched calibration solutions prepared in a synthetic groundwater matrix to account for potential matrix effects. The main analytical figures of merit, including linearity, sensitivity, precision, accuracy, and preconcentration efficiency, are summarized in Table 5.

As shown in Table 5, the method exhibits good linearity over the concentration ranges of 0.025–50 μg L^−1^ for Pb(II) and 0.015–50 μg L^−1^ for Co(II), with correlation coefficients (R^2^) of 0.997 and 0.992, respectively. These values indicate reliable linear responses under matrix-matched conditions. The method provides low detection limits, with LOD/LOQ values of 0.08/0.24 μg L^−1^ for Pb(II) and 0.06/0.15 μg L^−1^ for Co(II), confirming the suitability of the dSPE procedure for trace-level determination using ICP–OES). The method showed satisfactory repeatability (RSD 2.3% for Pb(II) and 3.1% for Co(II)) and acceptable accuracy, with recoveries of 90.5% and 87.6%, respectively, indicating effective extraction under matrix-matched conditions. The experimentally obtained preconcentration factors (9.51 for Pb(II) and 9.23 for Co(II)) were close to the theoretical value of 10, reflecting efficient analyte retention and elution during the dSPE process.

### 3.4. Method Validation and Application to Real Samples

#### 3.4.1. Validation Using Certified Reference Material

The reliability of the proposed dSPE-based method was validated using NIST CRM 1643d (Trace Elements in Water), which contains certified concentrations of Pb and Co. The measured concentrations (Table 6) showed close agreement with the certified values, providing quantitative recoveries and acceptable precision. At the 95% confidence level, no statistically significant differences were observed between the found and certified values, confirming the method’s accuracy for trace determination in aqueous matrix.

#### 3.4.2. Analysis of Real Water Samples

The applicability of the developed method was further assessed using real water samples following extraction/preconcentration under the optimized dSPE conditions. Sample blanks were analyzed to account for potential reagent contributions, and the samples were spiked with Pb(II) and Co(II) at 5 and 10 µg L^−1^. As summarized in Table 7, recoveries for Co(II) ranged from 94 to 101% with RSD values in the ranges (2.23–3.6%), while Pb(II) recoveries ranged from 96 to 104% with RSDs of 2.08–3.65%. The wastewater analysis results (Table 7) further confirm the practical applicability of the method and its agreement with reference values reported in the literature [11].

### 3.5. Reusability and Stability

**Figure 10:**
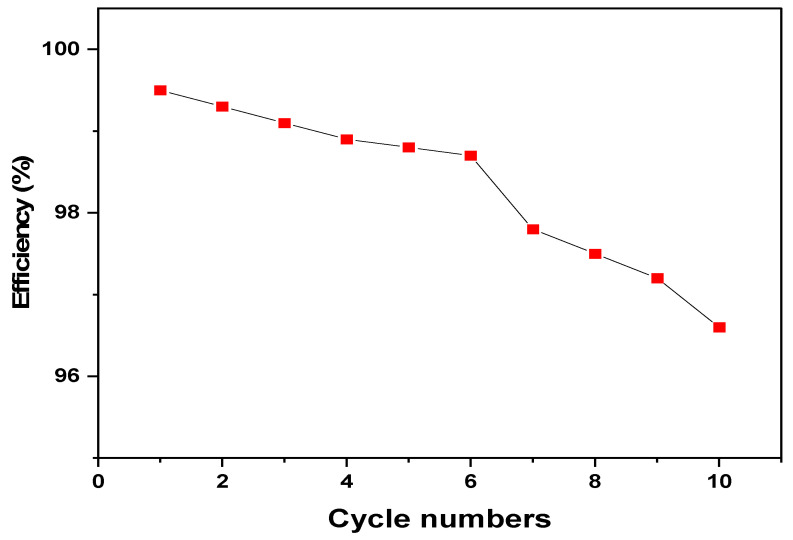
Relative sorption capacity of DSA-MSNs over ten adsorption–desorption cycles, showing minimal decline during the first seven cycles and a gradual drop thereafter.

The reusability of the DSA-MSNs was examined through ten sequential adsorption–desorption cycles, and the relative sorption capacity was measured after each cycle [10]. As shown in Figure 10, the retention profile exhibited minor fluctuations during the first seven cycles, with capacities remaining above 98.8% of the initial value. The drops during these early cycles were slow and within the normal variation expected from repeated washing and handling of the sorbent. A more noticeable decline appeared after cycle 7, where the capacity gradually decreased, reaching approximately 96.6% of the initial value after the tenth cycle. Despite this slight downward trend, the overall loss remained below 3.5%, indicating that the structural integrity of the fibrous KCC-1 framework and the stability of the immobilized Schiff-base ligand were largely preserved throughout reuse. This behavior confirms that the material maintains its functional performance across repeated dispersed SPE operations.

### 3.6. Comparison of the Performance of DSA-KCC-1 with Some SPE Sorbents

Table 8 compares the analytical performance of the DSA-KCC-1–based d-SPE method with representative SPE and d-SPE sorbents reported for Co(II) and Pb(II) determination [11,20,23,53,54,55,56,57,58,59,60,61]. The comparison shows that analytical sensitivity is primarily governed by nanostructured sorbent design and surface coordination chemistry, rather than by maximization of the preconcentration factor. The DSA-KCC-1 nanosorbent enables sub-μg L^−1^ detection limits for both Co(II) (0.06 μg L^−1^) and Pb(II) (0.08 μg L^−1^) with good repeatability (RSD ≈ 3%) using ICP-OES. These values are comparable to those reported for ICP-MS analysis following SPE using chelators like AXAD-4-PAR and PAC-MSNs [20,53], and are markedly superior to many FAAS- and AAS-coupled with silica, polymer, resin, and carbon-based sorbents [11,23,54,55,56,57,58,59,60]. Although lower LODs have been achieved using ICP-MS-hyphenated with d-SPE using nanosorbent [61], the present results indicate that efficient metal–ligand coordination on the dendritic fibrous KCC-1 surface enables high sensitivity even with less complex detection.

The data in Table 8 therefore position DSA-KCC-1 within the performance range of advanced SPE sorbents, highlighting the role of accessible fibrous morphology and tailored surface functionality in trace metal determination [11,20,23,53,54,55,56,57,58,59,60,61].

## 4. Conclusions

A fibrous silica-based chelating sorbent, DSA-KCC-1, was successfully synthesized and evaluated as a dSPE extractant for trace Pb(II) and Co(II) in aqueous samples. The dendritic fibrous KCC-1 framework provides high dispersibility and readily accessible binding sites, while covalent immobilization of DSA via Schiff-base formation introduces well-defined chelating functionality. Structural and surface characterization confirmed effective ligand grafting without disruption of the characteristic fibrous morphology. Adsorption studies showed rapid uptake capability for the studied metals, with equilibrium attained within 60 min. The adsorption kinetics followed a pseudo-second-order model, indicating chemisorption-controlled uptake, whereas equilibrium data were best described by the Langmuir isotherm, consistent with monolayer adsorption on homogeneous sites. These findings support a chelation-dominated adsorption mechanism. When applied as a sample-preparation sorbent in dSPE mode prior to ICP-OES analysis, the method enabled effective matrix separation and analyte enrichment, affording low detection limits, good linearity, and satisfactory precision. The method was validated using NIST SRM 1643d and successfully applied to the determination of Pb(II) and Co(II) in spiked groundwater and tap water samples.

## Figures and Tables

**Table 1:** Elemental analysis (EA) data of DSA–KCC-1 compared with calculated values for the proposed surface-grafted Schiff-base structure.

Element	Experimental Value	Calculated Value *
C (wt%)	9.38	9.46
H (wt%)	1.31	1.23
N (wt%)	0.61	0.61
C/N (mol/mol)	17.93	18.00
H/N (mol/mol)	28.84	28.00
Organic content (wt%)	≈11.9	≈12.0

* Calculated assuming 1:1 condensation of 3,5-Di-tert-butylsalicylaldehyde with aminopropyl-functionalized KCC-1 to form a surface-bound Schiff base.

**Table 2:** Textural properties of pristine KCC-1 and DSA-KCC-1 according to the BET and BJH equations represent the surface area (m^2^ g^−1^), pore volume (cm^3^ g^−1^), and pore diameter (nm), respectively.

Sample	BET Surface Area (m^2^/g)	Pore Volume (cm^3^/g)	Most Probable Pore Diameter (nm)
KCC-1	530	1.02	2.85
DSA-KCC-1	380	0.67	2.53

**Table 3:** Kinetic adsorption models, equations, and fitted parameters for Pb(II) and Co(II) adsorption onto DSA-KCC-1 at 298 K.

Model	Model Equation	Parameter	Pb(II)	Co(II)
Pseudo-first-order (PFO)	$q_{t}=q_{e}\left(1-e^{-k_{1}t}\right)$	$q_{max}$ (mg g^−1^)	50.41	42.96
		$k_{1}$ (min^−1^)	0.028	0.210
		RMSE	0.85	1.35
Pseudo-second-order (PSO)	$q_{t}=\frac{q_{e}^{2}k_{2}t}{1+q_{e}k_{2}t}$	$q_{max}$ (mg g^−1^)	52.13	45.04
		$k_{2}$ (g mg^−1^ min^−1^)	0.012	0.0091
		RMSE	0.77	0.56
Elovich	$q_{t}=\frac{1}{b}ln(1+\alpha bt)$	α (mg g^−1^ min^−1^)	$1.07\times 10^{6}$	$6.91\times 10^{3}$
		b (g mg^−1^)	0.34	0.28
		RMSE	2.18	2.09
Intraparticle diffusion (IPD)	$q_{t}=k_{\mathrm{id}}\;t^{1/2}+C$	$k_{\mathrm{id}}$ (mg g^−1^ min^−1^/^2^)	4.54	4.81
		C (mg g^−1^)	29.67	29.67
		RMSE	2.91	0.65

Definitions of Kinetic Model Terms: uptake at time t; $q_{e}$: equilibrium uptake; $q_{max}$: model capacity; $k_{1},k_{2}$: rate constants; α,b: Elovich constants; $k_{\mathrm{id}}$: diffusion constant; C: boundary layer. RMSE: model fit error.

**Table 4:** Adsorption isotherm models, equations, and fitted parameters for Pb(II) and Co(II) adsorption onto DSA-KCC-1 at 298 K.

Model	Model Equation	Parameter	Pb(II)	Co(II)
Langmuir	$q_{e}=\frac{q_{max}K_{L}C_{e}}{1+K_{L}C_{e}}$	$q_{max}$ (mg g^−1^)	99.73	66.26
		$K_{L}$ (L mg^−1^)	0.100	0.109
		RMSE	3.62	2.05
Freundlich	$q_{e}=K_{F}C_{e}^{1/n}$	$K_{F}$ (mg g^−1^)(g L^−1^)^1/*n*^	21.65	17.24
		n(–)	3.24	3.73
		RMSE	11.83	7.44
Redlich–Peterson (R–P)	$q_{e}=\frac{K_{RP}C_{e}}{1+\alpha_{RP}C_{e}^{\beta }}$	$K_{RP}$ (L g^−1^)	8.65	6.77
		$\alpha_{RP}$ ((mg L^−1^)^*g*^)	0.06	0.09
		β(–)	1.08	1.02
		RMSE	3.71	2.13

Definitions of Adsorption isotherm terms $:q_{e}$: equilibrium uptake; $q_{max}$: monolayer capacity; $K_{L}$, $K_{F}$, $K_{RP}$, $\alpha_{RP}$: isotherm constants; n, β: heterogeneity factors. RMSE: model fit error.

**Table 5:** Figures of merit and analytical characteristics of the proposed dSPE method for Pb(II) and Co(II) determination under matrix-matched conditions.

Analytical Parameters	Pb(II)	Co(II)
Linearity, μg/L	0.025–50	0.015–50
Correlation coefficient, R^2^	0.997	0.992
Limit of detection (LOD, μg/L)	0.08	0.06
Limit of quantification (LOQ, μg/L)	0.24	0.15
Average relative standard deviation (RSD %)	2.3	3.1
Average recovery (%)	90.5	87.6
Theoretical preconcentration factor (PF)	10	10
Actual preconcentration factor (PF)	9.51	9.23

**Table 6:** Validation of the proposed method using NIST CRM 1643d (Trace Elements in Water): certified and found concentrations of Co(II) and Pb(II), with recoveries.

Metal Ions	Certified (μg L^−1^)	Found (μg L^−1^)	Recovery (%)
Co(II)	25.05 ± 0.17	24.75± 0.9	98.80
Pb(II)	18.303 ± 0.081	18.12 ± 0.41	99.00

**Table 7:** Spike–recovery results for Pb(II) and Co(II) in real water samples after extraction/preconcentration using the optimized dSPE procedure.

Sample	Added (μg/L)	Found (μg/L)		Recovery (%)	
		Pb(II)	Co(II)	Pb(II)	Co(II)
Groundwater	-	1.12 ± 0.43	0.63 ± 0.07	-	-
	5	4.9 ± 0.15	4.8 ± 0.11	98	96
	10	9.6 ± 0.2	9.4 ± 0.21	96	94
Tap water	-	0.6	0.12	-	-
	5	5.2 ± 0.19	5.05 ± 0.17	104	101
	10	9.9 ± 0.31	9.7 ± 0.25	99	97

**Table 8:** Comparison of the proposed analytical method with other SPE-based methods for extraction and determination of the metal ions.

Ions	System	Method	PF	LOD/μg L^−1^	Detection	RSD (%)	Ref.
Co(II)	AXAD-4-PAR	SPE		0.053	ICP-MS	1.61	[53]
	Silica- and polymer	SPE	200	12.3	FAAS	1.3	[54]
	Silica- and polymer	SPE	260	3.4	FAAS	1.3	[55]
	PAC-MSNs	SPE	-	0.005	ICP-MS	1.89	[20]
	MCM-41	SPE	10.3	0.03	AAS	2.4	[11]
	Functionalized silica	SPE	200	0.2	FAAS	2.3	[56]
	Amberlite XAD-2	SPE	100	5	FAAS	≤8%	[57]
	DSA-KCC-1	d-SPME	9.23	0.06	ICP-OES	3.1	This work
Pb(II)	Activated carbon	SPE	120	1.7	FAAS	1.2	[58]
	Multi-walled carbon nanotubes (TETA- MWCNT)	SPE	113	3.7	AAS	1.4	[23]
	carbon-based sorbents	-	100	2.6	-	<5	[59]
	magnetic graphene oxide (GO@Fe_3_O_4_)	MSPE	167	0.35	FAAS	2.4	[60]
	Activated carbon-PTA	d-SPME	-	0.033	ICP-MS	1.29	[61]
	DSA-KCC-1	d-SPME	9.51	0.08	ICP-OES	3.2	This work

## Data Availability

Data available upon request from corresponding author.

## Abbreviations

The following abbreviations are used in this manuscript:

KCC-1	KAUST Catalysis Center-1
DSA	3,5-di-tert-butylsalicylaldehyde
BET	Brunauer–Emmett–Teller
BJH	Barrett–Joyner–Halenda
ICP-OES	Inductively Coupled Plasma Optical Emission Spectroscopy
DSA-KCC-1	3,5-di-tert-butylsalicylaldehyde –functionalized fibrous mesoporous silica
CTAC	Cetyltrimethylammonium chloride
D-SPE	Dispersive solid-phase extraction
EDS/EDX	Energy-dispersive X-ray spectroscopy
FESEM	Field-emission scanning electron microscopy
FTIR	Fourier transform infrared spectroscopy
LOD	Limit of detection
LOQ	Limit of quantification
LLE	liquid–liquid extraction
APTES	3-Aminopropyltriethoxysilane
qmax	Maximum adsorption capacity
SDA	Structure-directing agent
SEM	Scanning electron microscopy
TGA	Thermogravimetric analysis
TEM	Transmission electron microscopy
XRD	X-ray diffraction
